# Neonatal Hearing Screening: Prevalence of Unilateral and Bilateral Hearing Loss and Associated Risk Factors

**DOI:** 10.7759/cureus.15947

**Published:** 2021-06-26

**Authors:** Susan Sabbagh, Marzieh Amiri, Maryam Khorramizadeh, Zahra Iranpourmobarake, Mansoureh Nickbakht

**Affiliations:** 1 Department of Medical Anatomy, School of Medicine, Dezful University of Medical Sciences, Dezful, IRN; 2 Musculoskeletal Rehabilitation Research Center, Ahvaz Jundishapur University of Medical Sciences, Ahvaz, IRN; 3 Department of Audiology, School of Rehabilitation Sciences, Ahvaz Jundishapur University of Medical Sciences, Ahvaz, IRN; 4 Department of Medical Physics, School of Medicine, Dezful University of Medical Sciences, Dezful, IRN; 5 Department of Speech Pathology, School of Health and Rehabilitation Sciences, University of Queensland, Brisbane, AUS

**Keywords:** hearing loss, neonatal, screening, prevalence, risk factor

## Abstract

Introduction: Newborn hearing screening is essential for early identification of hearing loss to decrease the adverse effects of hearing loss. The objective of this study was to determine the prevalence of hearing loss and risk factors of congenital hearing loss in newborns.

Methods: In this analytical case-control study, a hearing screening test was performed for all newborns aged 3-14 days.

Results: Of 5,500 newborns evaluated, 24 newborns had hearing loss. The prevalence of hearing loss was 4.36 per 1,000. Of 24 hearing-impaired newborns, 15 had bilateral hearing loss (BHL) (62.5%) and nine had unilateral hearing loss (UHL) (37.5%). Among the neonates with hearing loss, the prevalence of hearing loss was higher (77.8%) in the right ear. The main risk factors identified in this study were low gestational age (*P*=0.001), hospitalization in the neonatal intensive care unit (NICU) (*P*=0.008), exposure to ototoxic drugs (*P*=0.001), gestational diabetes *P*=0.01), craniofacial anomalies (*P*=0.01), convulsion (*P*=0.03), consanguineous marriage of parents (*P*=0.001), low birth weight (*P*=0.01), and hyperbilirubinemia (*P*=0.001).

Conclusion: The prevalence of hearing loss was higher in the right ear than in the left ear. NICU stay, use of ototoxic drugs, low gestational age (<35 weeks), gestational diabetes, craniofacial anomalies, convulsion, consanguineous marriage of parents, low birth weight, and hyperbilirubinemia were significant risk factors for congenital hearing loss in studied newborns.

## Introduction

Hearing loss is one of the most prevalent sensory impairments in the world [1], with 34 million children being affected by this condition [2]. According to the World Health Organization (WHO), 466 million people have been diagnosed with hearing loss worldwide, and this number will increase to over 900 million by 2050 [3]. Therefore, early identification and intervention of hearing loss are important as the delayed diagnosis of hearing loss may affect early childhood development, including speech, language, and cognitive skills [4].

In the United States, it is estimated that bilateral hearing loss (BHL) occurs in one to two per 1,000 live births [5], and unilateral hearing loss (UHL) occurs one per 1,000 or approximately one-third of all newborns with congenital hearing loss [6]. Although children with UHL can hear with one normal ear, research showed that they had worse verbal cognitive and oral language scores compared to children with normal hearing [7]. In addition, recent studies revealed that each ear is structured to differentiate between various types of sounds and send them to the optimal side of the brain. It seems that the left ear provides extra amplification for tones (e.g., music), while the right ear provides extra amplification for rapid sounds like a speech [8]. It means that enhanced processing of tones occurs in the right auditory cortical areas while enhanced processing of rapid stimuli occurs in the left side that shows strong crossed connections from ears to central auditory processing pathways [8].

It was also shown that newborns had larger transient otoacoustic emissions (TEOAEs) (i.e., stimulated by clicks presented in a rapid manner) in the left ear and larger distortion product otoacoustic emissions (DPOAEs) (i.e., evoked by pairs of continuous tones) in the right ear [8]. These findings indicate that processing at the level of the ear may facilitate lateralization of auditory function in the brain [8].

Several studies have shown a number of risk factors for congenital hearing loss, including birth weight less than 1,250 grams, prematurity, hypoxia, Apgar score less than three and six in 1 and 5 minutes, respectively, seizures, ventilation, meningitis, jaundice, asphyxia, hypoglycemia, and treatment with aminoglycosides and furosemide. However, there are some newborns with hearing loss and without any known risk factors [9-11]. Therefore, hearing screening of all newborns is necessary to avoid negative outcomes of hearing loss for the children and their families.

Early intervention for neonates with congenital hearing loss requires early diagnosis and a suitable intervention within six months of birth. Evidence shows that hearing loss is the second most common disability in Iran [12]. There is also limited studies exploring the prevalence of neonatal hearing loss in Khuzestan Province, Iran. So, the aim of this study was to determine the prevalence of BHL and UHL, to identify the most critical risk factors of hearing loss, and to determine the degree of hearing loss in each ear of newborns in the north of Khuzestan Province.

## Materials and methods

Study design

This cross-sectional, case-control study was approved by the Ethics Deputy of Ahvaz Jundishapur University of Medical Sciences (IR.AJUMS.REC.1399.859) and carried out in accordance with the Declaration of Helsinki. All evaluated newborns were born in the north of Khuzestan Province, Iran, and admitted to the central health center in this area.

Exclusion criteria

Newborns with atresia or stenosis of one or both external ear canals and those without parental consent were excluded.

Study procedure

At first, a questionnaire was completed for all newborns, aged three to 14 days. A professional audiologist conducted hearing screening tests and filled the questionnaire (appendix). The collected data included demographic characteristics, family history of hearing loss, gestational diabetes, neonatal convulsion, hyperbilirubinemia, NICU stay, duration of hospitalization, craniofacial anomalies, newborn or mother's treatment with ototoxic drugs during intrauterine growth time or after birth (drugs including gentamicin, amikacin, tobramycin, furosemide, and vancomycin), parents’ consanguineous marriage, low birth weight (<2,500 g), and gestational age <35 weeks.

After giving verbal information about the hearing screening process to the parents, the informed consent forms were obtained from the parents before the hearing screening was carried out. All neonates were tested in a quiet room and the order of hearing tests was as below:

1. Otoscopy for checking the bilateral external auditory canals.

2. TEOAEs by Madsen Accuscreen (Madsen; Natus Hearing & Balance; Taastrup, Denmark): The intensity of click stimulation in the device was set to 35 dBnHL, and disposable ear tips were used to cover the probes during the test. When the test was done and there was an otoacoustic emission (OAE) response, the results were displayed as "PASS" (6 dB signal-to-noise ratio at least in three frequencies from 1,000 to 4,000 Hz) on the screen and if there was no response to a stimulus, as "REFER.”

3. Automated auditory brainstem response (AABR) tests by Madsen Accuscreen (Madsen; Natus Hearing & Balance; Taastrup, Denmark): The negative electrode was placed on the ipsilateral mastoid, the positive electrode was placed on the upper part of the forehead and the common electrode was placed at the contralateral mastoid. The impedance level was kept between 1 and 5 kΩ for the electrodes. Click stimuli at 35 dBnHL, with alternating polarity and presentation rate of 37.7/s, were used. No sedatives were used for this test.

Newborns who had abnormal results in one of the tests or one of the ears were reported as refer. Newborns with refer results were tested after two weeks as below:

1. Neonates were checked by a tympanometer with a high-frequency probe tone (1000 Hz) (Impedance Audiometer AZ 26, Interacoustics, MN, USA).

2. Diagnostic auditory brainstem response (ABR) was performed by click stimuli and presentation rate of 21.1/s with rarefaction polarity (Vivosonic Inc., Ontario, Canada). Wave V was traced for determining the auditory threshold.

3. Auditory steady-state response (ASSR) was performed for determining the degree of hearing loss (Vivosonic Inc., Ontario, Canada). This test was performed for octave frequencies from 500 to 4,000 Hz simultaneously for both ears.

As this study was a case-control study, the neonates with refer result, considered case group and a matched sex neonate from those who passed the screening stage, considered the control group. To reduce the selection bias of case-control, we used random methods when selecting neonates from populations. So, the selection was done by a staff of the central health center.

Statistical analysis

Data were analyzed using SPSS version 16 (SPSS Inc., Chicago, IL, USA). Mann-Whitney U test was performed for non-parametric variables, such as gender, gestational diabetes, craniofacial anomalies, NICU stay, use of ototoxic drugs, convulsion, and hyperbilirubinemia. The Student’s t-test was applied for parametric variables, including birth weight and gestational age. A P-value less than 0.05 was considered statistically significant.

## Results

A total of 5,500 newborns were screened in this study, including 2,970 (54%) males and 2,530 (46%) females. Of these, 190 (3.45%) infants failed in the screening and entered into the next stage. These infants selected as the case group and including 106 (55.8%) males and 84 (44.2%) females. From 5,310 newborns who passed the test, 190 matched sex newborns were randomly selected as the control group.

ABR test was carried out in the case group; 24 of whom were found to have a hearing loss. Given that the total number of newborns examined in this study was 5,500, the prevalence of hearing loss was 4.36 per 1,000 newborns.

Of 24 hearing-impaired newborns, 15 had BHL (62.5%) and nine had UHL (37.5%). Among the neonates with ULH hearing loss, the prevalence of hearing loss in the right ear was higher (77.8 %) than in the left ear (22.2%). The frequency distribution of severity of hearing loss was shown in Table 1.

**Table 1:** Prevalence of severity of hearing loss (n=24).

Degree of hearing loss	Number	Frequency (%)
Mild hearing loss (20-39 dB)	3	12.5
Moderate hearing loss (40-55 dB)	5	20.8
Severe hearing loss (70-90 dB)	9	37.5
Profound hearing loss (>90 dB)	7	29.2
Total	24	100

A significant difference between the case and control group was seen in the risk factors that were studied (Table 2).

**Table 2:** Comparison of risk factors between case (n=190) and control (n=190) groups. *related to Mann–Whitney U test.

	Case, N (%)	Control, N (%)	Z-value*	T-test	Significance
Birth weight per grams (SD)	2,620 g (0.64)	3,160 g (1.3)		2.43	P=0.01
Gestational age <35 weeks	57 (30%)	7 (3.6%)		6.23	P=0.001
Gestational diabetes	17 (8.9%)	5 (2.6%)	2.34		P=0.01
Craniofacial anomalies	24 (12.7%)	2 (1.1%)	2.1		P=0.01
Days in the intensive care unit ≥ 5 days	18 (9.4%)	4 (2.1%)	2.63		P=0.008
Use of ototoxic drugs	6 (3%)	1 (0.5%)	9.04		P=0.001
Hyper bilirubinemia	4 (2.1(%)	1 (0.5%)	4.13		P=0.001
Convulsion	10 (5.2%)	1 (0.5%)	2.15		P=0.03
Consanguineous marriage of parents	56 (29.8%)	34 (18%)	2.05		P=0.01

## Discussion

In this cross-sectional case-control study, we investigated the prevalence of hearing loss, risk factors, and the degree of hearing loss in newborns of north of Khuzestan in a 12-month period. We found that the prevalence of hearing loss was 4.36 per 1000 live births, which is consistent with the findings of Firouzbakht et al. [13] and Arjmandi et al. [14] who reported a prevalence of 4.7 and 4.8 per 1,000 live births in Iran, respectively.

The results of the present study demonstrated that the prevalence of hearing loss was higher in the right ear than the left ear. Consistent with our findings, Lieu et al. also reported that hearing loss in the right ear is more common than the left ear [7]. Misic et al. hypothesized that the right auditory cortex is better integrated into the connectome and facilitating more efficient communication with other areas [15]. In addition, recent studies revealed that each ear is structured to differentiate between various types of sounds. It seems that the left ear provides extra amplification for tones (e.g., music), while the right ear provides extra amplification for rapid sounds like speech. It means that enhanced processing of tones occurs in the right auditory cortex while enhanced processing of rapid stimuli occurs in the left auditory cortex that shows strong crossed connections from ears to central auditory processing pathways. Indeed, children with right-sided deafness might be more susceptible to learning loss than those with left-sided hearing loss [8]. In addition, it seems that the right ear is critical for learning [16]. Further investigation is required to determine whether there is a significant difference in hearing loss between the right and left ears. These findings are important as they can help us determine whether children with right ear hearing loss should receive different interventions, such as intensive early language therapy and amplification [7].

Another interesting finding in this study is the significant relationship between the use of ototoxic drugs and the higher occurrence of UHL. This finding is similar to Hirose et al.’s study that found medications, such as antibiotics and chemotherapy agents, could cause pediatric UHL. They mentioned that, although the causes of UHL are not well-known, the toxic effects of aminoglycoside antibiotics on cochlear hair cells have been identified as a risk factor [17]. However, the impact of ototoxic treatment on hearing loss development in newborns is controversial [10].

In the present study, the effects of environmental and demographic risk factors on hearing loss were also examined. Our findings revealed a significant association between low gestational age (<35 weeks) and hearing loss. In other studies also gestational age below 36 weeks has been considered as a risk factor for hearing loss [18]. Underdevelopment of the respiratory system and weakened immune system of premature neonates may expose them to various infection and as a result, the chance of sepsis will be raised [19]. 

We also found a significant correlation between low birth weight and hearing loss in newborns. It was reported that newborns with low birth weight had a high risk of hearing loss, so routine audiological assessments were recommended in these newborns. Although very low birth weight may not strongly influence the auditory system, it is usually associated with other risk factors, which can affect hearing synergistically. Therefore, the risk of hearing loss is significantly higher in newborns with low birth weight compared to the general population of newborns [20].

Another risk factor of hearing loss which is significantly different between the two studied groups was consanguineous marriage of parents. Lotfi et al. reported that in 45.7% of Iranian children with hearing loss, there was a history of consanguineous marriage of parents [21]. A review study by Shearer et al. also reported that approximately 80% of congenital and perilingual hearing losses are due to genetic causes [22]. So, before consanguineous marriage, genetic consultation is highly recommended.

Positive family history of hearing loss was another risk factor identified in the current study. The results of our study showed that the prevalence of hearing loss among families with a positive history of hearing loss was 13 per 1000 live births, which is probably due to the high rate of consanguineous marriage among people in the north of Khuzestan Province. However, a study in Turkey found that a family history of hearing loss is not a risk factor for ear anomalies in neonates [23]. The contradictory results of different studies highlight the need for further investigations in this area.

The presence of craniofacial anomalies was different between the case and control groups, and a significant relationship was found between these anomalies and hearing loss which is similar to other studies which reported that the anomalies are associated with hearing loss [4, 11]. Another finding of the present study was that newborns with convulsions after birth were significantly more likely to have hearing loss, which is in line with the study by Ohl et al. [4] who reported an association between hearing loss and convulsion, as a risk factor in newborns. Additionally, hyperbilirubinemia was found to be a risk factor for hearing loss in this study, which is in agreement with the findings reported by other studies on newborns with hearing loss [10,11]. In fact, high serum levels of unconjugated bilirubin can cross the blood-brain barrier and be induced ototoxicity within the brainstem and cranial nerve VIII, resulting in auditory neuropathy spectrum disorder [11].

The present findings of this study also found that the newborns of mothers with gestational diabetes were at a higher risk of hearing loss. Padmadasan and his colleagues reported the prevalence of deafness in neonates of mothers with diabetes mellitus was 7 times higher than the prevalence of this loss in neonates without any risk factor [24]. However, a systematic review reported contradictory results regarding neonatal hearing loss in the presence of gestational diabetes during pregnancy [25]. Because of the high energy utilization of the inner ear and due to microangiopathic processes that follow glucoprotein deposition caused by hyperglycemia, it is possible that gestational diabetes affects the auditory system [26]. Therefore, prospective cohort studies are needed to control the confounding factors in the correlation between gestational diabetes and hearing loss and to minimize measurement bias.

Furthermore, hospitalization in NICU for more than five days was another risk factor, significantly correlated with hearing loss. This finding is in agreement with some previous research [9, 10]. Bielecki et al. in a study of 5,282 infants, found that hospitalization in NICU in excess of seven days was a risk factor of hearing loss. They mentioned that increasing the number of risk factors an infant is exposed, will increase the probability of hearing loss [9]. Zamani et al. in a study of 905 infants who were hospitalized in NICU found that need to be in this section more than five hours may be considered a risk factor for hearing loss [22]. So, being in NICU must be considered a risk factor inducing neonatal hearing loss.

ABR and OAE tests are valuable screening tests, as they can be implemented within a short period after birth. However, it is important to notice that the results of OAE test can be influenced by the outer and middle ears, producing false-positive results. Some conditions, such as auditory neuropathy spectrum disorders (ANSDs) and inner hair cell dysfunctions, cannot be detected by OAE tests too; and false-negative results may be obtained [27]. Automated OAE tests may also indicate false passes in neonates with brain damage [10]. Young et al. reported that about 30% of newborns who had cochlear implantation, passed the hearing screening at birth [28]. Therefore, in our study, newborns were tested by both OAE and AABR tests to achieve a more accurate result.

This study has some implications for policymakers and clinicians. Firstly, the prevalence of hearing loss is still high so developing effective interventions to address the risk factors and prevent hearing loss in newborns is necessary. The interventions could be raising awareness of clinicians and expectant mothers and about potential risk factors of hearing loss by social media and educational programs. Second, clinicians should be trained to avoid prescribing ototoxic drugs for newborns if possible and inform parents about the potential risks of ototoxic drugs for their children.

## Conclusions

The prevalence of hearing loss in the current study was 4.36 per 1,000 newborns, which is consistent with the previous studies. We also found that the prevalence of hearing loss in the right ear was higher than in the left ear, which is an important finding as it can help us determine whether children with right ear hearing loss should receive different interventions or not. But further investigation is required to determine this.

NICU stay, use of ototoxic drugs, low gestational age (<35 weeks), gestational diabetes, craniofacial anomalies, convulsion, the consanguineous marriage of parents, low birth weight, and hyperbilirubinemia were significant risk factors for congenital hearing loss in our study population. It can be concluded that the use of TEOAE and AABR tests simultaneously is essential for the best diagnosis of congenital hearing losses. Therefore, timely detection and consider all risk factors of congenital hearing loss for appropriate next interventions is highly recommended.

## Appendices

No: …….

Sex: Female…….  Male……………

Date of birth: ………………….

Risk factors of hearing loss:

Family history of hearing loss: Yes ……….   No ……….

 Gestational diabetes: Yes ……….   No ……….

 Neonatal convulsion: Yes ……….   No ……….

 Hyperbilirubinemia: Yes ……….   No ……….

 NICU stay: Yes ……….   No ………. (duration of hospitalization: ……… days)

 Craniofacial anomalies: Yes ……….   No ……….

 Newborn or mother's treatment with ototoxic drugs during intrauterine growth time or after birth (drugs including gentamicin, amikacin, tobramycin, furosemide, and vancomycin): Yes ……….   No ……….

 Parents’ consanguineous marriage: Yes ……….   No ……….

 Low birth weight (<2,500 g): Yes ……….   No ………. Pease write the child weight: ………….

 Gestational age <35 weeks: Yes ……….   No ……….

Does child have any anomaly in his/her ear canal (such as atresia or stenosis): Yes ……….   No ……….

Audiological results:

Screening results:

TEOAEs: Pass………. Refer ……….

AABR: Pass………. Refer ……….

Diagnostic results:

ABR: Normal ……… Abnormal……… (Degree of hearing loss: ……………)

ASSR: Normal ……… Abnormal……… (Degree of hearing loss: ……………)

Tympanometry: Normal ……… Abnormal………
